# Activated CAMKKβ-AMPK signaling promotes autophagy in a spheroid model of ovarian tumour metastasis

**DOI:** 10.1186/s13048-020-00660-5

**Published:** 2020-05-11

**Authors:** Jeremi Laski, Bipradeb Singha, Xu Wang, Yudith Ramos Valdés, Olga Collins, Trevor G. Shepherd

**Affiliations:** 1 The Mary & John Knight Translational Ovarian Cancer Research Unit, Lawson Health Research Institute, London, ON Canada; 2 Departments of Anatomy & Cell Biology, Schulich School of Medicine & Dentistry, Western University, London, ON Canada; 3 West China School of Medicine, Chengdu, Sichuan China; 4 Departments of Oncology, Schulich School of Medicine & Dentistry, Western University, London, ON Canada; 5 Departments of Obstetrics & Gynaecology, Schulich School of Medicine & Dentistry, Western University, London, ON Canada; 6 London Regional Cancer Program, 790 Commissioners Rd. E., Room A4-836, London, ON N6A 4L6 Canada

**Keywords:** High-grade serous ovarian cancer, Spheroid, AMPK, Autophagy, CAMKKβ, STO-609

## Abstract

**Background:**

A hallmark of epithelial ovarian cancer (EOC) metastasis is the process of spheroid formation, whereby tumour cells aggregate into 3D structures while in suspension in the peritoneal cavity. EOC spheroids are subjected to bioenergetic stress, thereby activating AMP-activated protein kinase (AMPK) signaling to enter a metabolically quiescent state, which can facilitate cell survival under nutrient-limiting conditions. Independently, we have also demonstrated that EOC spheroids induce autophagy, a process that degrades and recycles intracellular components to restore energy and metabolites. Herein, we sought to examine whether AMPK controls autophagy induction as a cell survival mechanism in EOC spheroids.

**Results:**

We observed a co-ordinate increase in phosphorylated AMPK and the autophagy marker LC3-II during EOC spheroid formation. Reduced AMPK expression by siRNA-mediated knockdown of *PRKAA1 and PRKAA2* blocked autophagic flux in EOC spheroids as visualized by fluorescence microscopy using the mCherry-eGFP-LC3B reporter. A complementary approach using pharmacologic agents Compound C and CAMKKβ inhibitor STO-609 to inhibit AMPK activity both yielded a potent blockade of autophagic flux as well. However, direct activation of AMPK in EOC cells using oligomycin and metformin was insufficient to induce autophagy. STO-609 treatment of EOC spheroids resulted in reduced viability in 7 out of 9 cell lines, but with no observed effect in non-malignant FT190 cell spheroids.

**Conclusions:**

Our results support the premise that CAMKKβ-mediated AMPK activity is required, at least in part, to regulate autophagy induction in EOC spheroids and support cell viability in this in vitro model of EOC metastasis.

## Introduction

Epithelial ovarian cancer (EOC) is the deadliest gynecologic malignancy in women in the developed world, and is responsible for over 70% of all diagnosed cases [1]. The high mortality rates from EOC is most commonly attributed to late-stage diagnosis since its symptoms are shared with those of generalized post-menopausal conditions. In addition, current diagnostic tests are limited to physical pelvic exams, trans-vaginal ultrasound and CA-125 serum tests, all of which have low sensitivity for detection of early disease [2]. Since most EOC cases present with late-stage disease consisting of extensive tumour burden and ascites, treatment requires aggressive surgical debulking procedures coupled with cytotoxic chemotherapy to reduce to minimal residual disease and delay disease progression. Nevertheless, rates of recurrence remain exceptionally high, with relapsed EOC often acquiring chemo-resistance [1]. As such, gaining further understanding of the mechanisms governing late-stage EOC progression and recurrence of chemo-resistant disease is of utmost importance in developing more effective therapeutics [3, 4].

During metastasis, EOC cells detach from the primary tumour site, disseminate within the peritoneal fluid of the abdominal cavity, then subsequently re-attach to new sites thereby forming secondary lesions. A unique hallmark of EOC metastasis lies in the process of multicellular spheroid formation thereby affording metastatic cells with enhanced survival and chemo-resistance [5], as well as increased capacity to re-attach and invade the peritoneum [6]. Previous work by our group demonstrated that EOC cells enter a quiescent state within spheroids [7], and they possess reduced metabolic activity with increased AMP-activated protein kinase (AMPK) signaling [8]. AMPK is a conserved serine/threonine heterotrimeric kinase complex acting as a bio-energetic stress sensor in nearly all mammalian systems, primarily to promote cell survival during starvation-like conditions [9]. Following nutrient deprivation, increased levels of adenosine monophosphate (AMP) and adenosine diphosphate (ADP) bind to the gamma subunit of the complex, cause an allosteric shift, and thereby facilitate AMPK phosphorylation at the threonine 172 residue (T172) on the catalytic alpha subunit. AMPK activity often acts as a major bioenergetic regulator to induce catabolic processes while concurrently down-regulating anabolic processes, however, its exact functions are often tissue specific [9].

In separate studies, we demonstrated that EOC spheroids upregulate macroautophagy (described here as autophagy) [10, 11], a lysosomal process allowing for the degradation and recycling of intracellular nutrients and damaged organelles [12]. Dual roles have been suggested for autophagy in mediating cancer progression: autophagy can serve tumour suppressive functions particularly during disease initiation, yet a large proportion of studies have demonstrated essential tumour-promoting effects of autophagy in late-stage disease [13]. Tumour cells undergoing cellular stress due to hypoxia, lack of nutrient availability, and during metastasis, hijack native autophagy functions to recycle their intracellular constituents ultimately providing temporary alternative sources of energy and nutrients.

As such, AMPK signaling can act as a key link between metabolism homeostasis and autophagy regulation. Although AMPK has several means by which to activate autophagy [14], its potential regulation of autophagy in EOC cells and spheroids has not been determined yet. Herein, we sought to examine whether AMPK signaling mediates autophagy induction in a spheroid model of EOC metastasis. Our results demonstrate that intact AMPK activity is required but not sufficient to promote autophagic flux in EOC spheroids. Treatment of EOC spheroids with the CAMKKβ inhibitor STO-609 potently blocks AMPK activity and autophagic flux leading to reduced cell viability.

## Materials and methods

### Cell culture

Work was conducted with several established ovarian cancer cell lines: CaOV3, OVCAR3, OVCAR4, OVCAR5, and OVCAR8 (ATCC), COV318 and COV362 (gift from Zia Khan, Western University), all of which are classified as high-grade serous [15, 16], and HeyA8 cells (ATCC). The iOvCa147-MA line was generated by subcutaneous injection of high-grade serous iOvCa147 cells [17] into immune-compromised female mice, isolation and dissociation of the resultant tumour, followed by intraperitoneal injection into subsequent female mice. Malignant ascites fluid was collected aseptically and returned to tissue culture to generate the iOvCa147-MA line. STR analysis was performed by the TCAG Facility (Hospital for Sick Children, Toronto ON) to confirm its identity with the original iOvCa147 cell line. The immortalized human fallopian tube secretory epithelial cell line FT190 [18] (gift from Ronny Drapkin, University of Pennsylvania) was used as a non-malignant cell line control. Cells were cultured in either DMEM/F12 (Invitrogen) for iOvCa147-MA, CaOV3, COV318, COV362, and FT190, or RPMI (Wisent) for OVCAR3, OVCAR4, OVCAR5, OVCAR8, and HeyA8, and supplemented with 10% fetal bovine serum (FBS) (Wisent). Cells were either grown under adherent conditions using tissue culture-treated plastic (Sarstedt) or in suspension using Ultra-Low Attachment (ULA) dishes (Corning) as performed previously [7].

### siRNA knockdown

RNA interference-mediated knockdown was achieved using Dharmacon siGenome SMARTpool reagents: Non-targeting control pool #2 (siNT, D-001206-14-05), *PRKAA1* (D-001206-14-05) *PRKAA2* (M-005361-02-0005). Cells were seeded into 6-well adherent plates at 300,000 cells/well for iOvCa147-MA, or 100,000 cells/well for OVCAR8; the following day siRNA (siNT, or equimolar *PRKAA1/2*) was transfected according to manufacturer’s instructions as performed previously [8]. Cells were incubated with transfection mixtures for 3 days after which the cells were trypsinized and seeded into ULA dishes for protein isolation and fluorescence microscopy at 48 h.

### Protein isolation

Adherent cells were washed with ice-cold phosphate-buffered saline (PBS) and placed in modified radioimmunoprecipitation assay (RIPA) lysis buffer as described previously [11]. Cells were subsequently scraped and left to lyse on ice for 30 min. After high-speed centrifugation, supernatant was collected for protein quantification by Bradford assay (BioRad) and stored at − 80 °C for subsequent use. For protein lysates from spheroids, cell suspensions were collected from ULA plates and centrifuged at 2400 rpm for 3 minutes. The cell pellet was washed with ice-cold PBS and placed in modified RIPA lysis buffer for 30 min on ice and continued as described above.

### Immunoblotting

Protein lysates were prepared at 30 μg per sample and resolved by 8 or 12% sodium dodecyl sulfate–polyacrylamide gel electrophoresis (SDS-PAGE). Gels were transferred to a polyvinylidene difluoride (PVDF) membrane and blocked with 5% w/v BSA in TBST (10 mM Tris–HCl, pH 8.0, 150 mM NaCl, 0.1% Tween 20). Membranes were subsequently incubated with protein-specific primary antibodies at a 1:1000 dilution in 5% BSA/TBST and incubated overnight at 4 °C. Following primary antibody incubation, membranes were incubated for 1 h at room temperature with a peroxidase-conjugated anti-rabbit or anti-mouse immunoglobulin G (1:10000 in 5% BSA/TBST). Protein detection was achieved through enhanced chemiluminescence using Luminata Forte (Millipore) and imaging was performed using the Chemidoc™ MP 7 System (BioRad). Densitometric analyses were subsequently performed using ImageLab™ software with tubulin used as a loading control.

### Antibodies and other reagents

Primary antibodies were used to detect threonine-172 phosphorylated AMPKα (40H9), total AMPKα (D63G4), p62 (2775S), LC3B (5114S) (Cell Signaling Technology, Danvers, MA), or tubulin (T9026; Sigma, Mississauga, ON). Secondary antibodies used were anti-rabbit horseradish peroxidase (HRP; NA934V Chicago, GE Healthcare) and anti-mouse HRP (NA931V Chicago, GE Healthcare). The following pharmacologic agents were used: Compound C (P5499) and Metformin (D150959-5G) from Sigma (Mississauga, ON), and STO-609 (15325) and Oligomycin (11342) from Cayman Chemical (Ann Arbor, MI) at concentrations indicated in the text.

### Generation of mCherry-eGFP-LC3B clones

OVCAR8 cells were plated into 6-well adherent plates at a density of 100,000 cells/well. Cells were transfected with the pBABE-puro-mCherry-eGFP-LC3B plasmid (gift from Jayanta Debnath, Addgene plasmid #22418), using Lipofectamine 2000 reagent according to the manufacturer’s protocol (MAN0009872, Invitrogen, Carlsbad Ca). Following 72 h, cells were placed in puromycin-supplemented media (1 μg/ml) for 72 h. Puromycin-resistant fluorescent clones were selected using cloning rings and expanded clonal populations expressing detectable fluorescence were chosen for subsequent experiments. The COV318 and FT190 cell lines were generated in an identical fashion as described above, however, following puromycin treatment, transfected cells were pooled in suspension conditions using ULA dishes and selected on the basis of high red fluorescence.

### Live-cell fluorescence microscopy

Phase contrast and fluorescence images of OVCAR8-, COV318- and FT190-mCherry-eGFP-LC3B cells were captured using a Leica DMI 4000B inverted fluorescence microscope. Fluorescent images were captured using GFP and Y3 filter cubes and merged images were generated using the Leica Application Suite (LAS). Alternatively, STO-609-treated OVCAR8 mCherry-eGFP-LC3B cells were seeded at 2000 cells/well in 96-well round-bottom ULA plates. Each well was imaged by phase contrast, GFP and RFP using the IncuCyte® ZOOM live-cell imaging system (Sartorius) at 3h intervals for a total of 14 days; time-lapse videos were generated using the IncuCyte® built-in software. All images and videos are presented in their original format with no adjustments in colour or exposure correction.

### Fluorescence quantification

Using Image J (Version 2.0.0-rc-69/1.52p), a region-of-interest (ROI) was circumscribed around each OVCAR8-mCherry-eGFP-LC3B spheroid (siNT- and si*PRKAA1/2*-transfected cells*)* using the phase contrast image as a template. The ROI was subsequently superimposed onto both the GFP and Y3 channel images where overall fluorescence intensity was measured in arbitrary units relative to overall spheroid area. Alternatively, GFP and RFP fluorescence, and signal overlap, were quantified on IncuCyte® ZOOM images of individual OVCAR8-mCherry-eGFP-LC3B spheroids (*n* = 10) using the built-in analysis software and measured from days 2 to 12 as masked area per image.

### Cell viability

Spheroids were collected by centrifugation at 2400 rpm for 3 min, washed with PBS, and centrifuged at 1000 rpm for 3 min. Pelleted spheroids were disaggregated using 50 μL of 0.25% Trypsin-EDTA incubated for 10 min at 37 °C followed by the addition of 50 μL FBS. Trypan Blue Reagent (Gibco) was added at a 1:1 ratio and cell counts were performed on the BioRad TC10™ automated cell counter.

### Statistical analysis

Graphs were generated using GraphPad Prism 8 (La Jolla, California) and data are expressed as mean ± SD. Student’s *t*-test and ANOVA with either Dunnett’s or Sidak’s multiple comparison test were performed using GraphPad Prism 8; all results were considered significant at *p* < 0.05.

## Results

### Coordinated AMPK activity and LC3-II processing during spheroid formation

We demonstrated previously that AMPK is activated in EOC spheroids to promote cytostasis [8]. In an independent study, our group generated evidence that autophagy is rapidly induced in EOC spheroids also, and autophagy is required to maintain cell viability in these structures. Thus, we now seek to connect the kinetics and requirement for AMPK signaling with autophagy activation in our in vitro spheroid model of EOC metastasis. Assessment of autophagic flux can be initially performed by measuring protein expression of both microtubule-associated protein 1A/1B-light chain (LC3) and p62 (sequestosome-1). Being recruited to autophagosomal membranes, LC3 is proteolytically cleaved at its C-terminus followed by lipidation to generate LC3-II, making it an excellent marker for monitoring the progression of autophagy [12]. Due to its ubiquitin-binding domain, p62 is known to function as a mediator protein, targeting ubiquitinated proteins to the autophagosomal membrane. Accumulation of p62 protein levels is indicative of reduced autophagic flux, whereas its decrease over time indicates sustained autophagy induction [12].

**Fig. 1:**
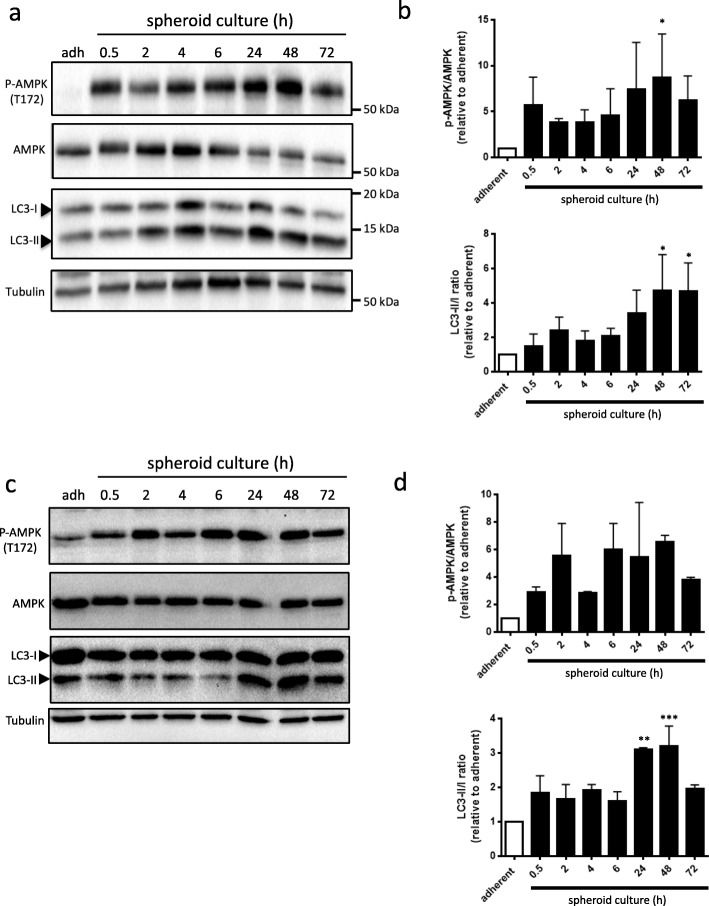
HGSOC spheroids have increased phosphorylated AMPK and LC3-II as compared with adherent cells. **a** iOvCa147-MA cells were trypsinized, seeded into ULA plates, and protein lysates were isolated at each time point as indicated. Adherent cell controls (adh) were cultured using standard tissue culture-treated plates for 72 h prior to protein isolation. Immunoblot analysis was performed for p-AMPK (T172), AMPK, and LC3B; tubulin served as a loading control. **b** Densitometric analysis of p-AMPK/AMPK and LC3-II:I ratio from the immunoblots were tested by one-way ANOVA followed by Dunnett’s multiple comparison test (*n* = 3) (*, *p* < 0.05). **c** OVCAR8 cells were trypsinized, seeded into ULA plates, and protein lysates were isolated at each time point as indicated. Adherent cell controls (adh) were cultured using standard tissue culture-treated plates for 72 h prior to protein isolation. Immunoblot analysis was performed for p-AMPK (T172), AMPK, and LC3B; tubulin served as a loading control. **d** Densitometric analysis of p-AMPK/AMPK and LC3-II:I ratio from the immunoblots were tested by one-way ANOVA followed by Dunnett’s multiple comparison test (n = 3) (*, *p* < 0.05)

To address AMPK phosphorylation kinetics and its relation to autophagy induction, iOvCa147-MA cells were seeded in ULA conditions and protein was isolated at various time points during spheroid formation. Following immunoblot analysis, we identified increased levels of LC3-II processing and increased phosphorylation of AMPK at T172 between 24h and 72 h relative to adherent cells (Fig. 1a&b). The highest levels of both p-AMPK and LC3-II was observed at 48 h; therefore, subsequent spheroid culture experiments were taken to the 48 h time point. Time course experiments conducted using OVCAR8 spheroids further confirmed the 48 h time point as optimal for evaluating AMPK activity and autophagy markers (Fig. 1c&d). Different levels of basal autophagy were observed in standard adherent conditions between the iOvCa147-MA and OVCAR8 cell lines, as we have seen previously among several ovarian cancer cell lines [11].

### AMPK knockdown inhibits autophagic flux in EOC spheroids but does not alter p62 or LC3 processing

**Fig. 2:**
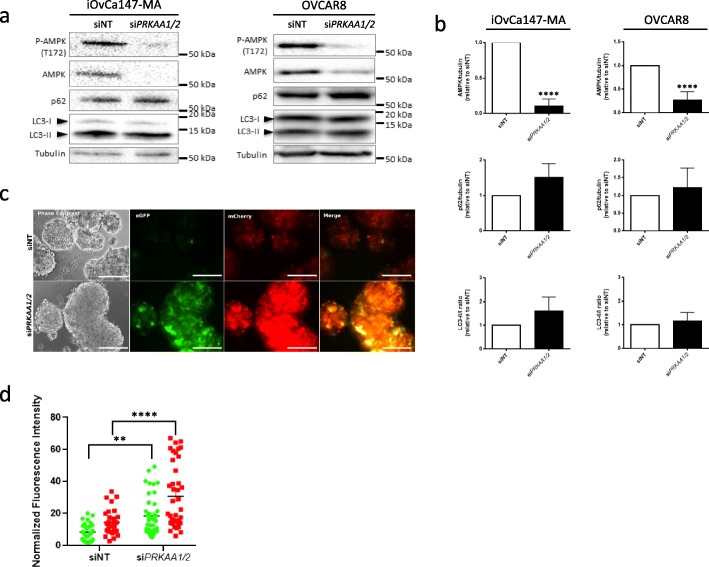
*PRKAA1/2* knockdown does not alter LC3-II and p62 levels in spheroids yet blocks autophagic flux. **a** Double knockdown of both AMPK α1 and α2 catalytic subunits was performed by co-transfection of *PRKAA1* and *PRKAA2* siRNA in adherent iOvCa147-MA and OVCAR8 cells; non-targeting siRNA (siNT) served as a control. At 72 h post-transfection, cells were trypsinized and seeded into 6-well ULA plates for 48 h. Immunoblot analysis was performed for p-AMPK (T172), AMPK, p62, and LC3B; tubulin served as a loading control. **b** Densitometric analysis for AMPK/tubulin, p62/tubulin, and LC3-II:I ratio from the immunoblots were tested for significance using a Student’s *t-*test (****, *p* < 0.001). **c** OVCAR8 mCherry-eGFP-LC3B cells were transfected with siNT and si*PRKAA1/2* as described above and seeded into 24-well ULA plates. Phase contrast and fluorescence images were captured at 48 h post-seeding. Scale bar = 200 μm. **d** Quantification of eGFP (green markers) and mCherry (red markers) fluorescence intensity per spheroid (normalized to spheroid area) in siNT and si*PRKAA1/2*-transfected OVCAR8-mCherry-eGFP-LC3B cells was performed using *Image J* software and tested for significance by two-way ANOVA followed by Sidak’s multiple comparison test (**, *p* < 0.01; ****, *p* < 0.001)

To elucidate the requirement of AMPK signaling regulation of autophagy in spheroids, we performed siRNA-mediated knockdown of the AMPK α1 and α2 catalytic subunits in iOvCa147-MA and OVCAR8 cells. AMPK exists as a heterotrimeric protein consisting of one catalytic α-subunit and two regulatory β- and γ-subunits. Although up to 12 different isomeric configurations are possible, there are only two known catalytic subunits encoded by the genes *PRKAA1* and *PRKAA2* [9]. Combined knockdown of *PRKAA1* and *PRKAA2* allowed us to control for variations in catalytic subunit expression and potential compensatory mechanisms, and to maximize AMPK attenuation. Following transfection in adherent conditions, cells were trypsinized and seeded into ULA conditions for 48 h, at which point protein was collected for immunoblot analysis. To our surprise, *PRKAA1/2* knockdown in iOvCa147-MA or OVCAR8 spheroids did not significantly alter LC3-II or p62 relative to siNT-transfected control spheroids (Fig. 2a&b). This was intriguing since AMPK has been implicated in several models as a canonical activator of autophagy, with its loss typically inhibiting autophagic flux [14, 19, 20]. No significant difference in spheroid cell viability was observed between the *PRKAA1/2* knockdown and siNT controls (data not shown), which corroborates the results from our previous study [8].

To further investigate the effect of *PRKAA1/2* knockdown on autophagic flux in EOC spheroids, we used OVCAR8 cells stably-transfected with an eGFP-LC3B reporter construct [10]. Following *PRKAA1/2 *knockdown, OVCAR8-eGFP-LC3B cells were seeded as spheroids and assessed using live-cell fluorescence microscopy. We observed a notable increase in green fluorescence in spheroids following *PRKAA1/2* knockdown indicating a block in autophagic flux (Figure S1). However, it is difficult to draw this conclusion, as well as adequately monitor autophagic progression from early-to-late stages, with a single fluorescence reporter construct. To address this issue, we stably transfected OVCAR8 cells with the dual fluorescence mCherry-eGFP-LC3B reporter [21]. Following autophagosome fusion with the acidic lysosome, the pH-sensitive eGFP signal is quenched, whereas the mCherry signal remains unaffected. Highly autophagic cells will exhibit predominantly red fluorescent punctae indicative of increased autophagic flux. Conversely, inhibiting autophagy induces an increase in green fluorescence due to reduced autophagosome fusion with lysosomes. Although this reporter has been used in adherent culture systems [21, 22], it can also be applied to spheroid models [23]. By placing OVCAR8 mCherry-eGFP-LC3B cells into ULA conditions and assessing overall fluorescence colour shift rather than individual autophagic punctae, we can characterize general autophagic flux within spheroids in a rapid manner.

*PRKAA1/2* knockdown in OVCAR8 mCherry-eGFP-LC3B spheroids resulted in a dramatic increase in green and red fluorescence relative to siNT-transfected control spheroids, which had predominantly low levels of fluorescence signal (Fig. 2c&d). To confirm our interpretation of a block in autophagic flux, we treated spheroids with chloroquine (CQ), a well-characterized lysosomotropic agent that inhibits lysosomal fusion to the autophagosome [12], and which we have demonstrated previously inhibits autophagy in EOC cells and spheroids [10, 11]. Treatment of OVCAR8 mCherry-eGFP-LC3B spheroids with 50 μM CQ for 4 h resulted in similar accumulation of green fluorescence as we observed with the *PRKAA1/2* knockdown (data not shown). Thus, *PRKAA1/2* knockdown can reduce autophagic flux in EOC spheroids; however, based on our immunoblot data, this observed AMPK-mediated regulation of autophagy may occur in an LC3- and p62-independent manner.

**Fig. 3:**
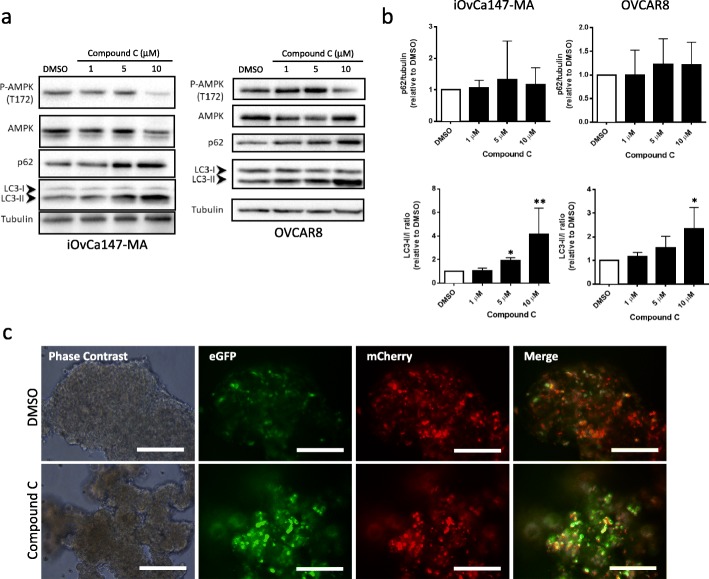
Pharmacologic inhibition of AMPK using Compound C increases LC3-II and blocks autophagic flux in spheroids. **a** iOvCa147-MA and OVCAR8 cells were seeded into 6-well ULA plates to form spheroids for 24 h prior to treatment with Compound C at the indicated concentrations; DMSO was the vehicle control. Protein lysates were isolated at 24 h post-treatment. Immunoblot analysis was performed for p-AMPK (T172), AMPK, p62, and LC3B; tubulin served as a loading control. **b** Densitometric analysis for p62/tubulin and LC3-II:I ratio from the immunoblots were tested by one-way ANOVA followed by Dunnett’s multiple comparison test. Asterisks indicate significant differences relative to control (*, *p* < 0.05; **, *p* < 0.01). **c** OVCAR8 mCherry-eGFP-LC3B cells were seeded into 24-well ULA plates, cultured for 24 h, then treated with 10 μM Compound C, or DMSO as a control, for an additional 24 h. Phase contrast and fluorescence images were captured. Scale bar = 200 μm

In addition to *PRKAA1/2* knockdown, we sought to examine the effect of a pharmacological inhibitor of AMPK on EOC spheroids. Currently, Compound C (also known as dorsomorphin) is the only known selective inhibitor of AMPK [24]. Treatment of both iOvCa147-MA and OVCAR8 cells with Compound C resulted in modest reduction of p-AMPK at 10 μM (Fig. 3a), yet significant increases in LC3 processing and a slight increase in p62 levels were observed (Fig. 3a&b). OVCAR8 mCherrry-eGFP-LC3B spheroids treated with 10 μM Compound C for 24 h exhibited a detectable increase in green fluorescence relative to their DMSO-treated controls (Fig. 3c).

### AMPK activation alone is insufficient to induce autophagy

We have shown previously that proliferating adherent EOC cells have relatively low levels of both autophagy and p-AMPK yet are robustly induced upon spheroid formation [8, 11]. As such, we deemed it important to test whether AMPK activity on its own is sufficient to induce autophagy in EOC cells. To achieve this, we activated AMPK by using the mitochondrial inhibitors oligomycin (100 nM) and metformin (2 mM), since both drugs are known to increase p-AMPK and its activity [25]. Oligomycin and metformin treatment for 24 h led to increased p-AMPK in adherent OVCAR8 cells, however no significant changes were observed in LC3-II processing or p62 levels (Figure S2). Taken together, our results suggest that AMPK activation is required in part for autophagic flux in EOC spheroids, yet on its own is insufficient to induce autophagy in adherent EOC cells.

### Pharmacologic inhibition of CAMKKβ reduces AMPK phosphorylation and inhibits autophagic flux

Due to the limited availability of small molecule inhibitors of AMPK, we sought to attenuate AMPK phosphorylation by targeting upstream kinases that lead to AMPK activation. Liver Kinase B1 (LKB1) encoded by *STK11*, is the best-characterized upstream kinase of AMPK. LKB1 is a highly-conserved serine-threonine kinase that typically functions as a regulator of cellular metabolism within the AMPK signaling axis [26]. Surprisingly, recent work from our laboratory identified that EOC spheroids lacking LKB1 expression by CRISPR-mediated *STK11* knockout sustain elevated p-AMPK suggesting alternative kinase(s) target AMPK in our system [27].

**Fig. 4:**
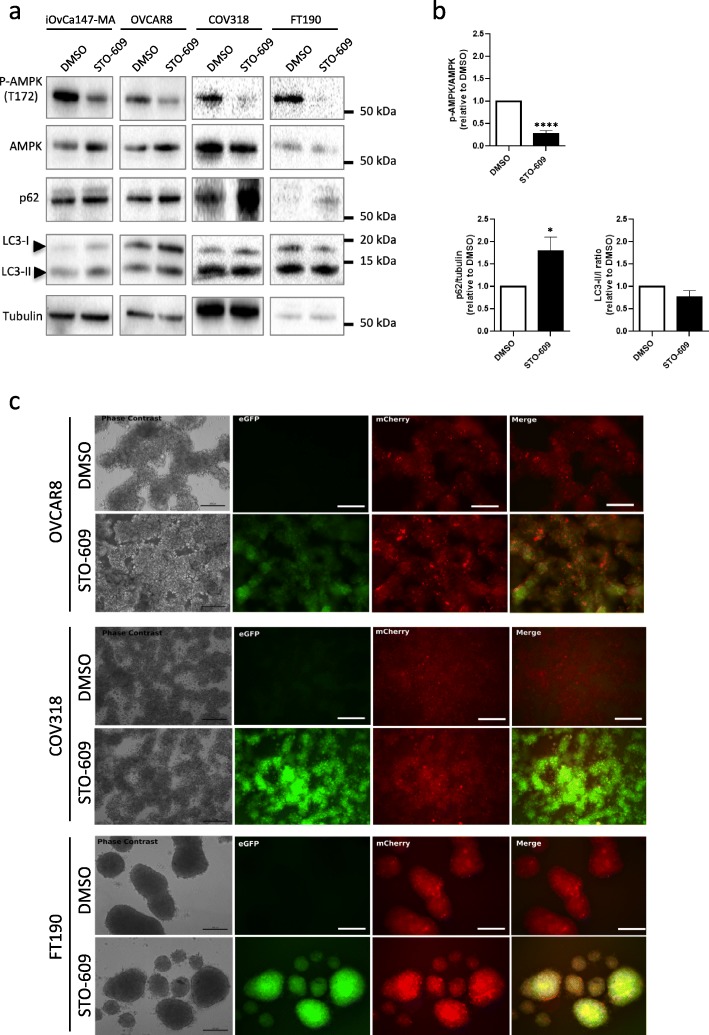
STO-609 treatment reduces p-AMPK, increases p62, and blocks autophagic flux in spheroids. **a** Cell lines (EOC cells: iOvCa147-MA, OVCAR8 and COV318; non-malignant cells: FT190) were seeded into 6-well ULA plates to form spheroids for 24 h prior to treatment with 10 μM STO-609, or DMSO as a vehicle control. Protein lysates were isolated at 24 h post-treatment. Immunoblot analysis was performed for p-AMPK (T172), AMPK, p62, and LC3B; tubulin served as a loading control. **b** Densitometric analysis for p-AMPK/AMPK, p62/tubulin and LC3-II:I ratio from the immunoblots for the three EOC cell lines together were tested by Student’s *t*-test. Asterisks indicate significant differences relative to control (*, *p* < 0.05; ****, *p* < 0.001). **c** Representative phase contrast and fluorescence images of DMSO- and STO-609-treated spheroids using OVCAR8-mCherry-eGFP-LC3B, COV318-mCherry-eGFP-LC3B, and FT190-mCherry-eGFP-LC3B cells. Treatments were performed as described above and images were captured after 24 h. Scale bar = 200 μm

Previous literature has implicated calcium/calmodulin-dependent protein kinase beta (CAMKKβ) as an alternative AMPK activating kinase [28]. For example, cellular matrix deprivation leads to CAMKKβ-mediated AMPK phosphorylation in breast cancer cell lines [29]. As such, we decided to use a selective CAMKKβ inhibitor, STO-609, as another method to attenuate AMPK phosphorylation. We included additional cell lines, the high-grade serous cancer COV318 cells, and immortalized human fallopian tube secretory epithelial FT190 cells. Treatment of non-malignant FT190 and EOC cell line spheroids with 10 μM ST0–609 resulted in significant reduction in p-AMPK (Fig. 4a). In addition, we observed a significant increase in p62, but no change in LC3-II levels (Fig. 4b). ST0–609 treated spheroids exhibited increased green fluorescence relative to their DMSO control indicating robust autophagic flux inhibition (Fig. 4c). Enhanced green fluorescence was observed in FT190 spheroids, suggesting that CAMKKβ-mediated regulation of AMPK and autophagic flux occur in both EOC cells and their potential premalignant precursor cells, too.

**Fig. 5:**
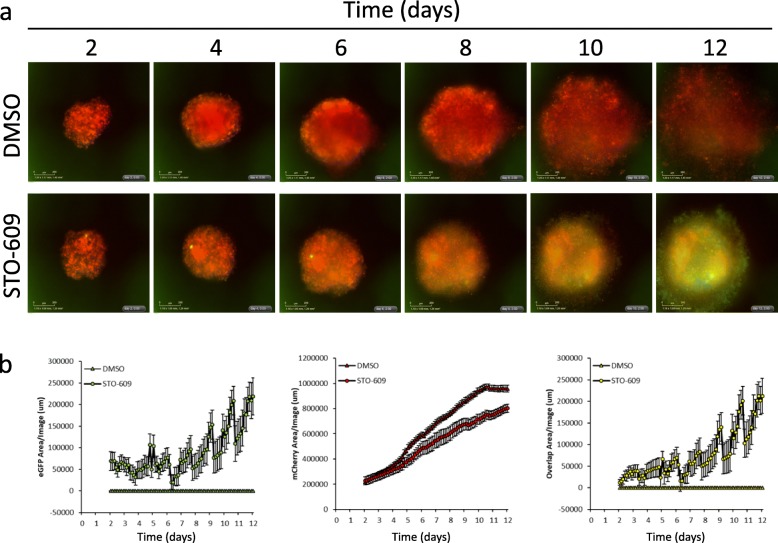
Time course of STO-609-mediated inhibition of autophagic flux in OVCAR8 mCherry-eGFP-LC3B spheroids. **a** OVCAR8 mCherry-eGFP-LC3B cells were pretreated with either 10 μM STO-609 or DMSO and seeded at a density of 2000 cells/well into 96-well round-bottom ULA plates. Images of eGFP and mCherry fluorescence signals were captured using the IncuCyte® ZOOM live-cell imaging system at 3h intervals over a period of 14 days. Representative images are shown for specific time points (days 2–12). Scale bar = 200 μm. **b** Quantification of eGFP, mCherry and Overlap fluorescence signals were quantified from 10 independent wells using the IncuCyte® ZOOM image analysis software. Data (mean ± SD) are displayed starting at day 2 to allow for complete aggregation of cells into individual spheroids, and to avoid the increased background fluorescence signal on image edges in the GFP channel at these early time points

**Fig. 6:**
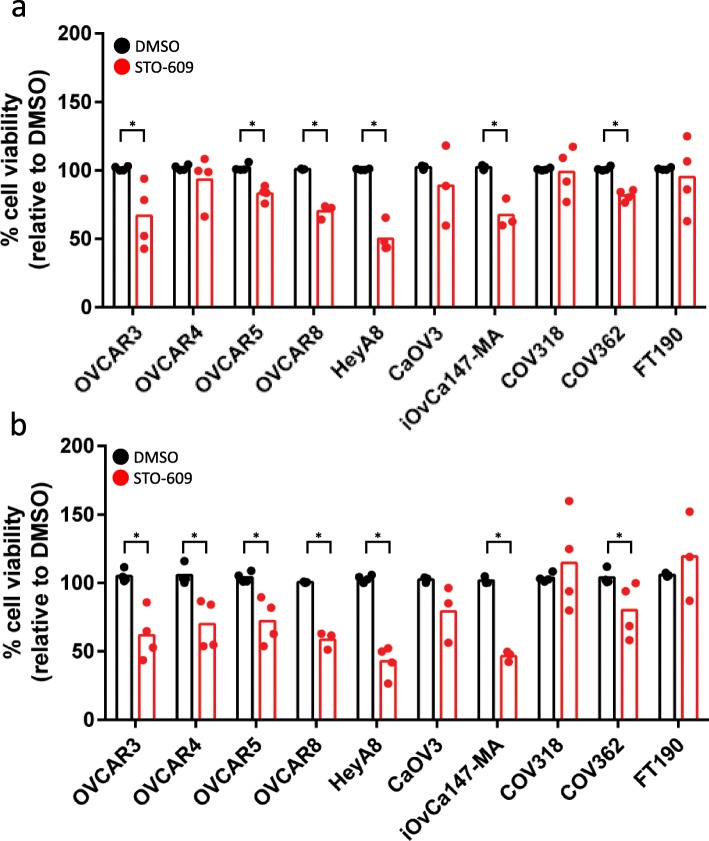
STO-609 treatment reduces spheroid viability across several EOC cell line spheroids. Cells were seeded into 24-well ULA plates and treated with 10 μM STO-609 or DMSO at the time of seeding. Trypan Blue Exclusion cell counting was performed at days 3 (**a**) and 6 (**b**); viability data was normalized to DMSO-treated controls set to 100%. Student’s *t*-test was performed to determine statistical significance for each cell line (*, *p* < 0.05)

We have demonstrated previously that autophagy is critical to maintain EOC cell viability in spheroids [10, 11], thus we postulate that potent inhibition of AMPK activity using STO-609 over an extended period would negatively impact EOC spheroid cell viability. First, we treated OVCAR8 mCherrry-eGFP-LC3B spheroids with 10 μM STO-609 or DMSO for up to 12 days to visualize extent of autophagic flux inhibition and spheroid integrity. Autophagic flux was potently blocked by STO-609 over the complete time period as evidenced by increased green fluorescence; we also observed a small decrease in overall spheroid size due to STO-609 treatment (Fig. 5a&b). Subsequently, we evaluated the effects of CAMKKβ-AMPK signaling inhibition by treating nine different high-grade serous EOC spheroids, and FT190 spheroid controls, with STO-609 for 3 and 7 days prior to quantifying viable cell number. After 3 days of STO-609 treatment, we observed a significant reduction in cell viability in 6 out of 9 EOC cell line spheroids (Fig. 6a); extending this treatment to 7 days resulted in an additional cell line (OVCAR4) sensitive to CAMKKβ-AMPK inhibition (Fig. 6b). Cell viability for two EOC cell lines, CaOV3 and COV318, as well as normal FT190 spheroids, was unaffected by STO-609 treatment at both time points. In summary, our results implicate CAMKKβ-mediated activation of AMPK is required for autophagy induction and resultant cell survival in EOC spheroids.

## Discussion

Dysregulation of autophagy has long been implicated in numerous pathologies [30]. In the context of metastatic ovarian cancer, it appears that autophagy serves a tumour protective role. We have demonstrated previously that EOC spheroids display increased levels of autophagy and that its inhibition can reduce overall EOC spheroid viability [10, 11]. Independently, we demonstrated that p-AMPK is increased in both patient-derived EOC spheroids as well as those generated in vitro, relative to proliferating adherent cells [8]. In this study, we sought to bridge these two phenomena and demonstrate that AMPK activity is required for autophagy induction in EOC spheroids to maintain cell viability.

In several biological contexts, AMPK activity on its own can lead directly to autophagy induction. However, treatment of adherent OVCAR8 cells with AMPK activators did not significantly change LC3-II or p62 expression. Metformin and oligomycin treatments increased p-AMPK levels, but these drugs function indirectly to activate AMPK by inhibiting mitochondrial respiration [31, 32]. Thus, caution must be taken when using these methods for AMPK activation and correlating results with autophagy induction. However, we consider it unlikely that AMPK activation is sufficient on its own to induce autophagy in proliferating adherent EOC cells.

We show that RNAi-mediated AMPK inhibition strongly inhibits autophagic flux as visualized by fluorescence microscopy. Interestingly, this phenotype may occur in an LC3- and p62-independent manner. The discrepancy in our results between fluorescence reporter and immunoblot assays raise certain questions as to what specific players mediate autophagy induction in ovarian cancer. Initially described in yeast as ATG8, several orthologs of the ubiquitin-like LC3 protein have been identified in mammals, although most work focuses on LC3B. More recently, studies have implicated LC3B-independent forms of autophagy. One candidate LC3 ortholog is gamma-aminobutyric acid receptor-associated protein (GABARAP). GABARAP has been shown to possess separate functions from LC3, as it is involved in late-stage autophagosome maturation [33]. More recently, LC3-independent autophagy in rat hepatocytes was shown to be regulated primarily through the GABARAP complex in the autophagosome [34]. Since we did not observe major effects on LC3 processing, we are currently investigating whether AMPK inhibition affects GABARAP expression and function in autophagic flux in EOC spheroids.

Although Compound C did not attenuate p-AMPK levels nearly to the same extent as either RNA interference or STO-609 treatment, it clearly inhibited autophagic flux in EOC spheroids. Compound C may affect autophagy as a combination of AMPK inhibition as well as with other potential targets of this agent. Previous literature identified multiple intersecting pathways that are potently affected by Compound C that are independent of AMPK (Harhaji-Trajkovic et al., 2010; Zhao et al., 2018). In fact, Compound C is known to affect BMP and mTOR signaling [24, 35]; we have demonstrated that both of these signaling pathways impinge upon the EOC spheroid phenotype [7, 10, 36]. As such, the combined action of Compound C on multiple different kinases could lead to our observed LC3 reporter results in EOC spheroids. In fact, we observed poor p-AMPK attenuation using Compound C, and it has conflicting roles as either an activator or inhibitor of autophagy [35]. Thus, use of this agent alone poses a limitation for analysis of AMPK regulation of autophagy in our system.

To address this, we present new findings regarding the requirement of CAMKKβ-mediated AMPK signaling in modulating autophagy in EOC. Treatment of EOC spheroids with the CAMKKβ inhibitor, STO-609, supports our *PRKAA1/2* knockdown data, thus strengthening the notion that AMPK is required for autophagy induction in EOC cells under spheroid conditions. This phenotype holds true not only for EOC cell lines, but also in non-malignant fallopian tube epithelial cells. Perhaps the autophagic stress response mediated by AMPK is conserved in secretory epithelial cells, as well as the high-grade serous EOC cells from which they arise.

Furthermore, work in our laboratory identified recently that LKB1-deficient EOC spheroids still retain the capacity to induce p-AMPK through CAMKKβ activity [27]. This finding together with our results herein suggest a crucial role for CAMKKβ in regulating p-AMPK levels in this disease. It has been previously reported that a rise in cytosolic calcium can induce autophagy through CAMKKβ in both MCF-7 and HeLa cell lines, highlighting an ATP-independent mechanism for autophagy induction [37]. More recently, cellular matrix deprivation has been identified as an inducer of intracellular calcium spikes, which in turn can activate AMPK through CAMKKβ signaling [29]. Examination of the calcium-oxidant signaling network in EOC spheroids might highlight a unique characteristic of these cancer cells that would lend itself to therapeutic inhibition. As such, it would be prudent to further characterize both the AMPK-dependent and -independent roles of CAMKKβ in the context of ovarian cancer. Our encouraging results of CAMKKβ-AMPK inhibition using the STO-609 and its negative impact on EOC spheroid cell viability lends even more support for such an intervention.

Overall, it appears that AMPK is required in part to induce autophagy in EOC spheroids, although this may occur in an LC3-and p62-independent manner. We also show AMPK phosphorylation is regulated by CAMKKβ activity in EOC spheroids to promote autophagic flux in these structures. These findings have contributed to our understanding of signaling axes regulating autophagy induction in EOC cells, and may represent novel therapeutic targets for this critical stress response in the setting of metastatic disease.

## Supplementary information

**Additional file 1: Figure S1.***PRKAA1/2* knockdown potentially inhibits autophagy in OVCAR8 eGFP-LC3B spheroids. Adherent cells were transfected with non-targeting siRNA (siNT) or si*PRKAA1/2,* or left untransfected, for 72 h. Cells were seeded into 24-well ULA culture dishes for 48 h prior to capturing phase contrast and fluorescence images. Scale bar = 200 μm.

**Additional file 2: Figure S2.** Pharmacologic AMPK activation does not alter LC3 processing and p62 levels in adherent iOvCa147-MA and OVCAR8 cells. (a) iOvCa147-MA and OVCAR8 cells were plated at a density of 150,000 cells/well in 6-well tissue-culture-treated plates and left to attach overnight. Cells were subsequently treated for 24 h with either Oligomycin (100 nM), or Metformin (2 mM, iOvCa147-MA; 1 mM, OVCAR8), or DMSO vehicle control. Immunoblot analysis was performed for p-AMPK (T172), AMPK, p62 and LC3B; tubulin served as a loading control. (b) Densitometric analysis of p62/tubulin and LC3-II:I ratio from the immunoblots were tested by one-way ANOVA followed by Dunnett’s multiple comparison test (*n* = 3) and no significant differences were observed.

## Data Availability

All data generated or analyzed during this study are included in this published article (and its supplementary information files). Materials used in this study are available from the corresponding author on reasonable request.
