# Supplementary figures and images for: Activated CAMKKβ-AMPK signaling promotes autophagy in a spheroid model of ovarian tumour metastasis
Source: J Ovarian Res. 2020 May 11;13:58. doi: 10.1186/s13048-020-00660-5 (PMC7216359; doi:10.1186/s13048-020-00660-5)

Figure S1

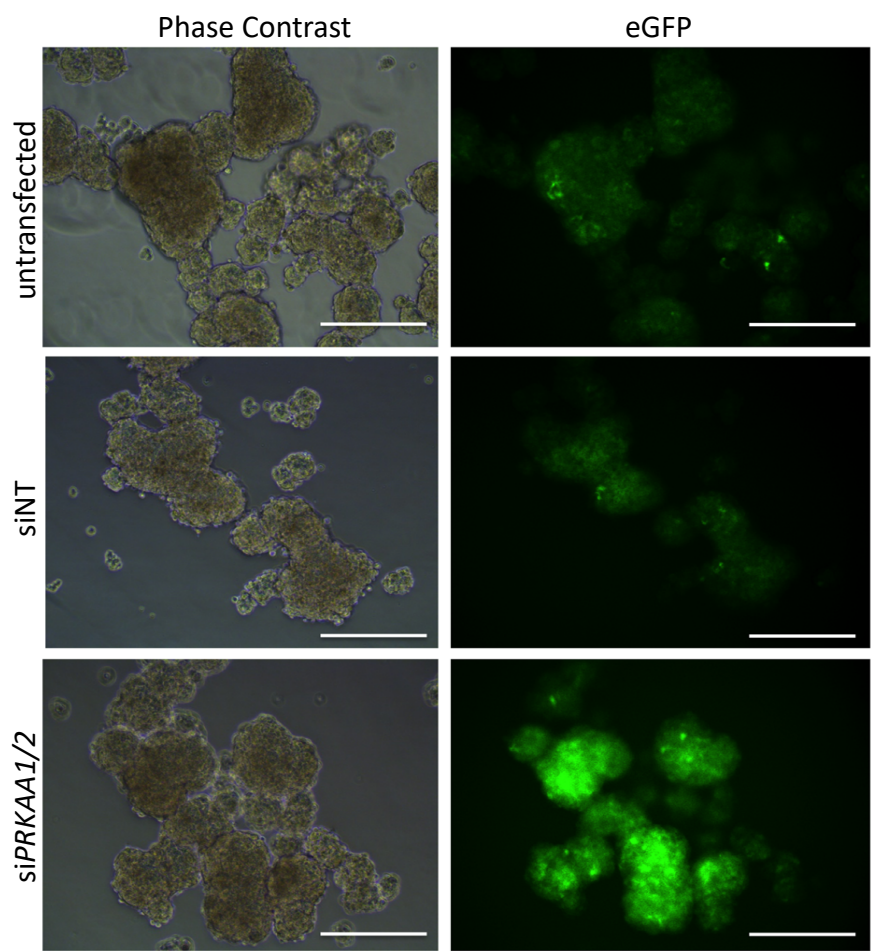

Supplement: Supplementary file 1 — Additional file 1: Figure S1.PRKAA1/2 knockdown potentially inhibits autophagy in OVCAR8 eGFP-LC3B spheroids. Adherent cells were transfected with non-targeting siRNA (siNT) or siPRKAA1/2, or left untransfected, for 72 h. Cells were seeded into 24-well ULA culture dishes for 48 h prior to capturing phase contrast and fluorescence images. Scale bar = 200 μm. [file 13048_2020_660_MOESM1_ESM.pdf]

Figure S2

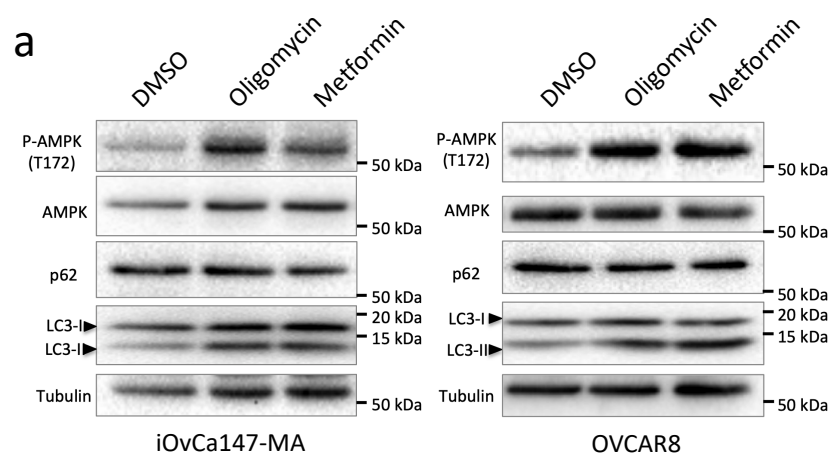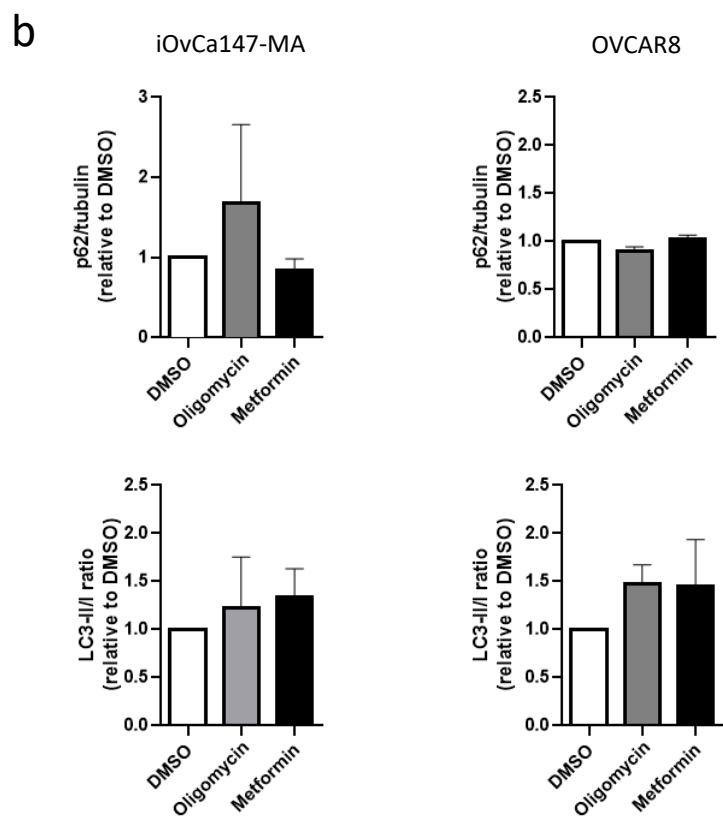

Supplement: Supplementary file 2 — Additional file 2: Figure S2. Pharmacologic AMPK activation does not alter LC3 processing and p62 levels in adherent iOvCa147-MA and OVCAR8 cells. (a) iOvCa147-MA and OVCAR8 cells were plated at a density of 150,000 cells/well in 6-well tissue-culture-treated plates and left to attach overnight. Cells were subsequently treated for 24 h with either Oligomycin (100 nM), or Metformin (2 mM, iOvCa147-MA; 1 mM, OVCAR8), or DMSO vehicle control. Immunoblot analysis was performed for p-AMPK (T172), AMPK, p62 and LC3B; tubulin served as a loading control. (b) Densitometric analysis of p62/tubulin and LC3-II:I ratio from the immunoblots were tested by one-way ANOVA followed by Dunnett’s multiple comparison test (n = 3) and no significant differences were observed. [file 13048_2020_660_MOESM2_ESM.pdf]
